# Supplementary material for: Mutation in the Kinase Domain Alters the VEGFR2 Membrane Dynamics
Source: Cells. 2024 Aug 13;13(16):1346. doi: 10.3390/cells13161346 (PMC11352761; doi:10.3390/cells13161346)
Supplement: Supplementary file 1 [file cells-13-01346-s001.zip › cells-3055743-supplementary.pdf]

Supplementary Figure S1

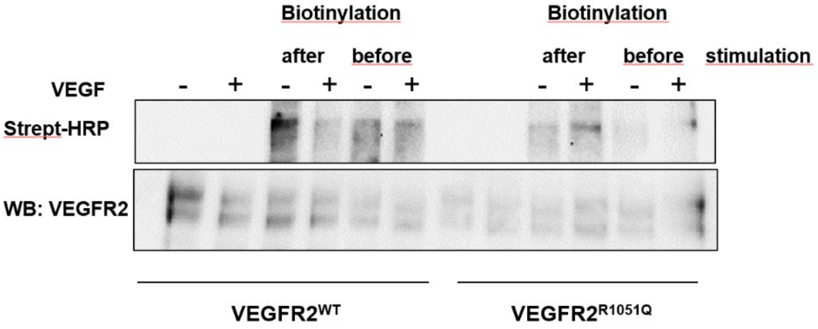

Supplementary Figure S1: VEGF- modulates the membrane exposure of VEGFR2^{R1051Q}: MCF7 cells were transfected with VEGFR2^{WT} or VEGFR2^{R1051Q} and biotynilated after or before VEGF-A treatment. Cell lysates were separated on 6%SDS-PAGE gel and blotted with streptavidin HRP to highlight VEGFR2 on cell membrane.
